# Genetic variation of dopamine and serotonin function modulates the feedback-related negativity during altruistic punishment

**DOI:** 10.1038/s41598-017-02594-3

**Published:** 2017-06-07

**Authors:** Sören Enge, Hendrik Mothes, Monika Fleischhauer, Andreas Reif, Alexander Strobel

**Affiliations:** 1 Department of Psychology, Faculty of Natural Sciences, MSB Medical School Berlin, Berlin, Germany; 2 Department of Psychology, Technische Universität Dresden, Dresden, Germany; 3 Department of Sports Science, University of Freiburg, Freiburg, Germany; 4 Brandenburg Medical School Theodor Fontane, Neuruppin, Germany; 5 PFH Private Hochschule Göttingen, Göttingen, Germany; 6 Department of Psychiatry, Psychosomatics and Psychotherapy, University Hospital Frankfurt/Main, Frankfurt/Main, Germany

## Abstract

Why do humans cooperate and often punish norm violations of others? In the present study, we sought to investigate the genetic bases of altruistic punishment (AP), which refers to the costly punishment of norm violations with potential benefit for other individuals. Recent evidence suggests that norm violations and unfairness are indexed by the feedback-related negativity (FRN), an anterior cingulate cortex (ACC) generated neural response to expectancy violations. Given evidence on the role of serotonin and dopamine in AP as well as in FRN-generation, we explored the impact of genetic variation of serotonin and dopamine function on FRN and AP behavior in response to unfair vs. fair monetary offers in a Dictator Game (DG) with punishment option. In a sample of 45 healthy participants we observed larger FRN amplitudes to unfair DG assignments both for 7-repeat allele carriers of the dopamine D4 receptor (DRD4) exon III polymorphism and for l/l-genotype carriers of the serotonin transporter gene-linked polymorphic region (5-HTTLRP). Moreover, 5-HTTLPR l/l-genotype carriers punished unfair offers more strongly. These findings support the role of serotonin and dopamine in AP, potentially via their influence on neural mechanisms implicated in the monitoring of expectancy violations and their relation to impulsive and punishment behavior.

## Introduction

In recent years, theoretical and empirical work has advanced knowledge about the behavioral and neuronal underpinnings of altruistic punishment (AP), a type of punishment behavior that is frequently expressed in the face of social norm violation, non-cooperation and unfairness. AP refers to the human tendency to punish norm violation of others with own costs but a potential benefit for others. As such, it has been shown to sustain a high level of cooperation in unrelated social groups and even in one-shot interactions^1–4^. It is assumed that AP may have evolved by gene-culture co-evolution^5^, thereby suggesting a critical role of genetic differences in AP. However, research on the role of genetic variation in AP is still limited e.g. refs 6, 7. The present study addresses this gap.

In experimental settings, AP behavior has been studied by economic games such as the Dictator Game (DG), the Ultimatum Game (UG) or by several modifications of both (e.g. refs 1, 8). Using such games, evidence on the neuronal correlates of AP has been gained^7, 9^. In a seminal functional magnetic resonance imaging (fMRI) study of Sanfey and colleagues, for example, increased activity in the anterior cingulate (ACC), the dorsolateral prefrontal cortex (DLPFC) and the insula for unfair versus fair monetary offers was found in an UG, with the insula predicting subsequent rejection of such offers^10^. It was argued that insula activation may be related to an emotional motivation to reject unfair offers, while DLPFC activation reflects a cognitive motivational signal that provides a bias toward acceptance, and that ACC activation is associated with the conflict between these two motivational signals. This concurs with a large body of evidence of fMRI and electroencephalographic (EEG) studies demonstrating that the ACC is critically implicated in performance monitoring and feedback processing, subserving behavioral adjustments in the face of conflict, erroneous performance or expectancy deviation (i.e., prediction error signals), respectively^11–13^. At the EEG level, accumulated evidence shows that the so called feedback-related negativity (FRN), an ACC-generated event-related potential (ERP) with a maximum around 300 ms at fronto-central electrodes is evoked following feedback about negative performance^14, 15^ or when outcomes are worse than expected^16^. In relation to economic decision making, larger (i.e., more negative-going) FRN amplitudes have been observed in response to unfair relative to fair offers in Ultimatum and Dictator games^17, 18^. Moreover, there is evidence that the FRN is related to rejecting unfair offers^19^ and is sensitive to utilitarian information indicating losses versus gains^20^.

Furthermore, knowledge on the underlying motives of AP has increased. As proposed by theoretical models^3^, AP is considered a social norm enforcing behavior, with internalized social norms being the assumed predictor of punishment (i.e., due to the violation of such norms). However, this notion has been increasingly challenged. It has been shown that impulsive-emotional processes elicited by unfair behavior or norm violations of others (e.g., anger, frustration, or provocation) can also trigger AP^21, 22^. Similarly, revenge-like motives appear to be constitutive for AP^9, 23^. In this context, a recent study of Brethel-Haurwitz and colleagues^24^ found no differences in punishment behavior between highly altruistic participants (kidney donors) and controls and concluded in suggesting that altruistic punishment may better be termed costly punishment to avoid the connotation that this behavior is predominantly driven by altruistic motives. In accordance with such findings, one of the few studies that addressed the impact of neuromodulators in AP showed that pharmacological manipulation of central serotonin availability via tryptophan depletion predicts AP^25^. This is supported by results of an impulsive choice task in the same study, akin to the critical role of serotonin in impulsivity and punishment^26, 27^. Follow-up studies seem to support the role of serotonergic modulation in both AP^28^, and also in FRN amplitude, as recently shown for a risk taking game using a molecular genetic variation of serotonin system function^29^. In this study, the 5-HTTLPR promoter polymorphism (i.e., the serotonin transporter gene linked polymorphic region) was addressed that functionally alters mRNA expression levels of the serotonin transporter gene, with lower transcriptional efficiency and lower serotonin transporter function in short (s) relative to long (l) allele carriers^30^. Indeed, serotonergic impact on impulsive and (altruistic) punishment behavior would match accumulated evidence demonstrating that differences in serotonergic signaling critically contribute to impulsivity-related phenotypes, traits and disorders^26, 31^ as well as to pro- and antisocial behavioral tendencies^32, 33^. Specifically, in terms of 5-HTTLPR, meta-analytic data suggest a relationship of homozygous l-allele carriers with impulsive phenotypes^34, 35^. In contrast, s-allele carriers are assumed to exhibit relatively higher scores in measures of anxiety, neuroticism, and harm avoidance^30, 36, 37^. Consistently, s-allele carriers have been shown to be more risk averse during economic decision making than those homozygous for the l-allele who are in turn more prone to risk taking tendencies that overlap with impulsivity^29, 38, 39^.

Similarly, there is evidence that individual differences in dopaminergic (DA) function may influence AP. Using a modified version of the DG, we found that genetic variation in the catechol O-methyltransferase (COMT) gene (*COMT* Val158Met) predicted higher punishment-related nucleus accumbens activation in Met-allele carriers that presumably exhibited higher synaptic dopamine availability^7^. Further support for the role of DA gene variations in economic decision making, pro-social behavior, and impulsivity is provided by a functional polymorphism in dopamine D4 receptor gene, the so-called *DRD4* exon III polymorphism. The 7-repeat allele of *DRD4* exon III is associated with a blunted efficacy to reduce cyclic adenosine monophosphate (cAMP) production as well as with decreased ligand binding^40^, and reduced D4 receptor expression^41^. Notably, the 7-repeat allele has been linked with approach- and impulsivity-related behavior and personality traits such as novelty seeking^42, 43^, impulsivity^44^, and financial risk taking^39, 45, 46^. Although there is only scarce meta-analytic evidence on the role of the *DRD4* exon III 7-repeat allele in impulsive personality traits^47, 48^, the 7-repeat allele has consistently been identified as a risk factor for another impulsive phenotype, i.e., attention-deficit/hyperactivity disorder^35, 49–51^. Furthermore, consistent with a seminal study of Bachner-Melman, *et al*.^52^, we found 7-repeat carriers to exhibit lower scores in self-reported altruism compared to non-carriers^53^, which concurs to other findings in the domain of prosocial behavior and economic decision making (for review, see ref. 54). However, as outlined above, the assumption that AP is predominantly driven by altruistic motives has not gone without criticism, while other factors mainly related to impulsive behavior (which is partly linked to the 7-repeat allele), are suggested to be constitutive for punishment behavior. Dopamine D4 receptors are preferentially expressed in prefrontal brain areas including the ACC, a main target of midbrain dopamine innervation^55, 56^. Therefore, genetic variations in the *DRD4* gene can be assumed to modulate ACC-mediated feedback processing and FRN amplitude^57^. In fact, it is proposed in FRN theory that FRN amplitude reflects an ACC-originated outcome of a prediction error that is linked to phasic responses of midbrain dopamine neurons during feedback processing. Specifically, in the model of Holroyd and Coles^14^ who adopted seminal research on animals and reinforcement learning theory^58^, it is suggested that both phasic increases and decreases in the subcortically located mesencephalic dopamine system are conveyed to the ACC when feedback is either better or worse than expected. Negative feedback (e.g., referring to aversive outcomes, punishment, or the cessation of reward) is assumed to disinhibit ACC neurons as a consequence of a phasic cessation of midbrain dopamine firing, in turn leading to markedly increased FRN amplitudes. Interestingly, in addition to the key role of DA signaling, recent theorizing also proposes a serotonergic contribution to negative prediction errors. This is due to its role in modulating aversive and punishment signals and in acting as a potential motivational opponent to DA signaling^26, 59^, thereby presumably affecting feedback processing and FRN amplitudes.

In the present study, we sought to examine *DRD4* exon III and 5-HTTLPR variations that impact on DA and 5-HT signaling as candidates to predict variability in FRN amplitude and AP behavior in response to unfair versus fair monetary dictator assignments. We used a modified DG that enabled to tap these responses from a first person and a third party perspective^7, 17, 38^. As far as we know, no study to date has examined the influence of allele-specific differences in *DRD4* exon III and 5-HTTLPR on the FRN component during AP. Due to their suggested relationship, we expected *DRD4* exon III 7-repeat allele carriers as well as homozygous l-allele carriers of 5-HTTLPR to show increased FRN amplitudes and stronger punishment behavior in response to unfair relative to fair monetary assignments of dictators.

## Methods

### Participants

All participants received written and oral information about the procedure and the aims of the study, gave written informed consent prior to the beginning of the study that could be withdrawn anytime without giving any reasons and were fully debriefed after completion. All data were collected and processed anonymously (pseudonymization). The procedure used in this study was in accordance with the Declaration of Helsinki (revised version) and formally approved by the ethics committee of the Technische Universität Dresden. The sample comprised 45 student volunteers (12 men; age MW = 22.4, SD = 3.8, range 15–34 years). All participants were of middle European ancestry and reported German as their mother tongue, had normal or corrected-to-normal vision and reported no relevant current health problems and no history of neurologic or psychiatric diseases, psychopharmacological treatment, and substance abuse/dependence. Data for the present study originate from a project examining the neurophysiological correlates of AP^17^.

### Genotyping

Buccal samples were obtained and DNA was extracted using the Oragene^TM^ DNA Self-Collection Kit (DNA Genotek Inc., Canada). *DRD4* exon III genotypes were determined as described earlier^41^. In line with previous studies^46^, participants with one or two copies of the 7-repeat allele were referred to as 7-repeat carriers (7R+; *n* = 16) and were compared with 7-repeat non-carriers (7R−; *n* = 28). 5-HTTLPR was genotyped according to a previously reported protocol^30^. Additionally, a functional single-nucleotide polymorphism (SNP) within the 5-HTTLPR l-allele was determined with an A to G substitution (rs25531). As in previous studies, homozygous s-allele, LG/LG and s/LG-cases were collapsed (e.g. ref. 60), and reclassified as s-allele carriers (N = 28). Carriers with two copies of the LA-allele are referred to as l/l genotype (n = 15). Genotypes of *DRD4* Exon III and 5-HTTLPR were in Hardy-Weinberg equilibrium (all *p* > 0.20).

### Procedure

The participants were seated in a dimly lit, acoustically shielded EEG cabin. They received written study information, gave their written informed consent and were instructed to complete a battery of questionnaires assessing their subjective financial situation (“How do you evaluate your current financial situation?” ranging from 1 = very favorable to 4 = very unfavorable; for better interpretation, the variable was recoded), their sleep duration as well as their nicotine, caffeine, and alcohol consumption during the previous 24 hours (see also ref. 17, for further information about questionnaires not included in this study). Then, participants received instructions for the subsequent EEG recordings and DG scenarios. Each experimental session consisted of two counterbalanced DG runs (first person perspective, third party perspective; see below) while the EEG was recorded. Before the DG started, participants completed 12 practice trials and they were encouraged to ask remaining questions. After the DG runs, each participant provided a saliva sample for genotyping.

### Dictator Game

**Figure 1:**
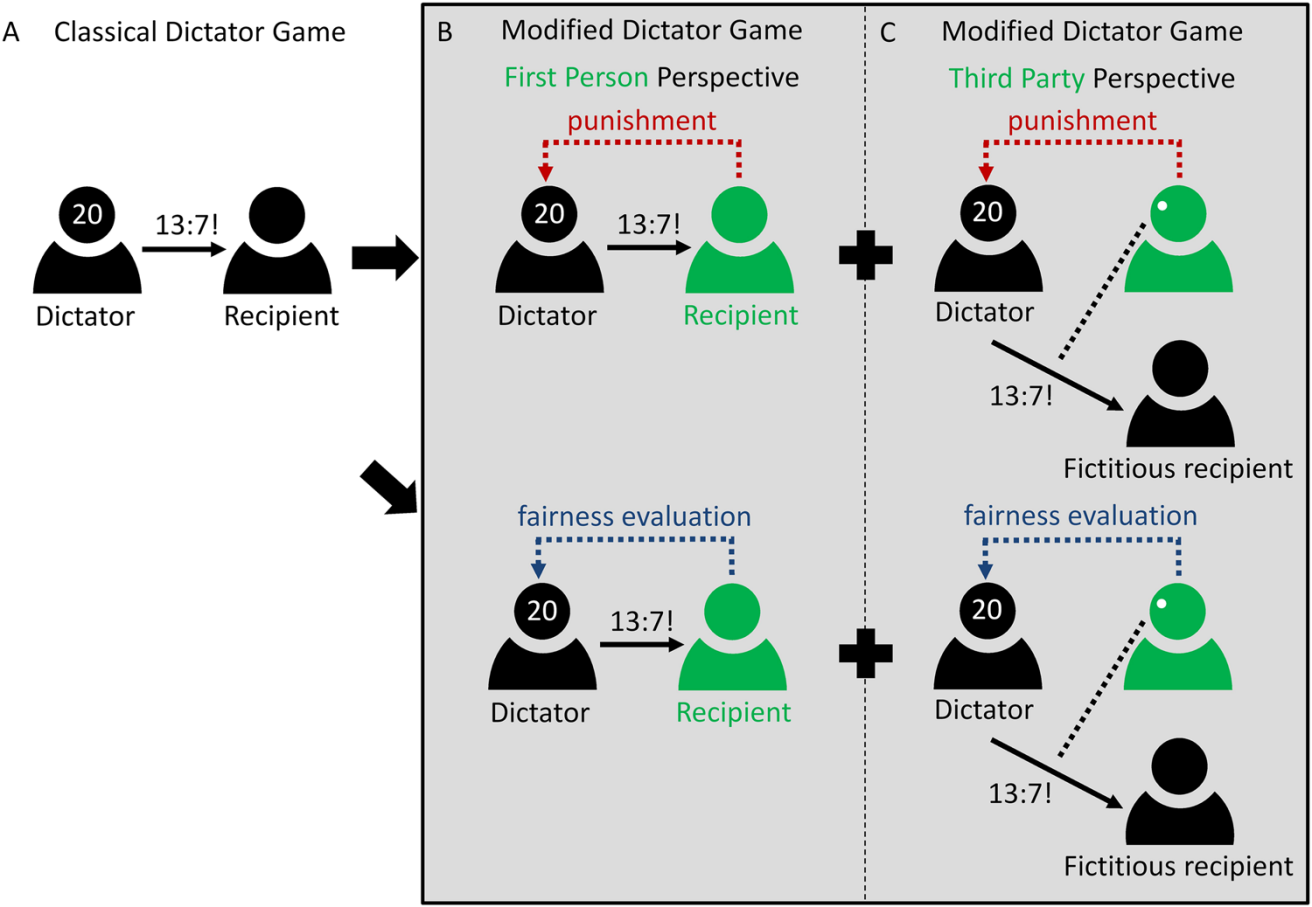
Illustration of different versions of the Dictator Game. (**A**) The classical Dictator Game. (**B**) and (**C**) Modified versions of the Dictator Game that were used in this study employing both a punishment option and a fairness evaluation from a first person perspective (**B**) and a third party perspective (**C**). Participants are symbolized in green. Solid arrows indicate dictator assignments, red dashed arrows indicate punitive acts, blue dashed arrows indicate fairness assessments.

A classical DG is an economic experimental game that represents an abstract social situation, in which one individual is given a certain amount of money (e.g., 20 €), whereas another individual is given nothing. The individual given the money (typically termed “the dictator”) has then the opportunity to share some amount of the received money (e.g., 7 €) at his or her discretion with the second individual (typically termed “the recipient”). The receiving individual has to accept this offer, as the name of the game suggests (see Fig. 1A). In line with previous research (e.g. ref. 7), we used a modified version of the DG with our participants being in the role of the recipient. Unlike the classical DG, in this modified DG recipients could be active in such a way that they still had to accept every dictator offer, but had the opportunity to either punish the dictator for an unfair assignment or to rate the fairness of the money split (depending on the condition, see Fig. 1B). Moreover, we not only examined allocations in which participants were directly affected by the dictator assignments, that is from a first person perspective (Fig. 1B), but also allocations from a third party perspective^7, 61^ in which participants only observed third, fictitious passive players receiving certain money splits, and then had to make their decision with respect to punishment or fairness (see Fig. 1C).

**Figure 2:**
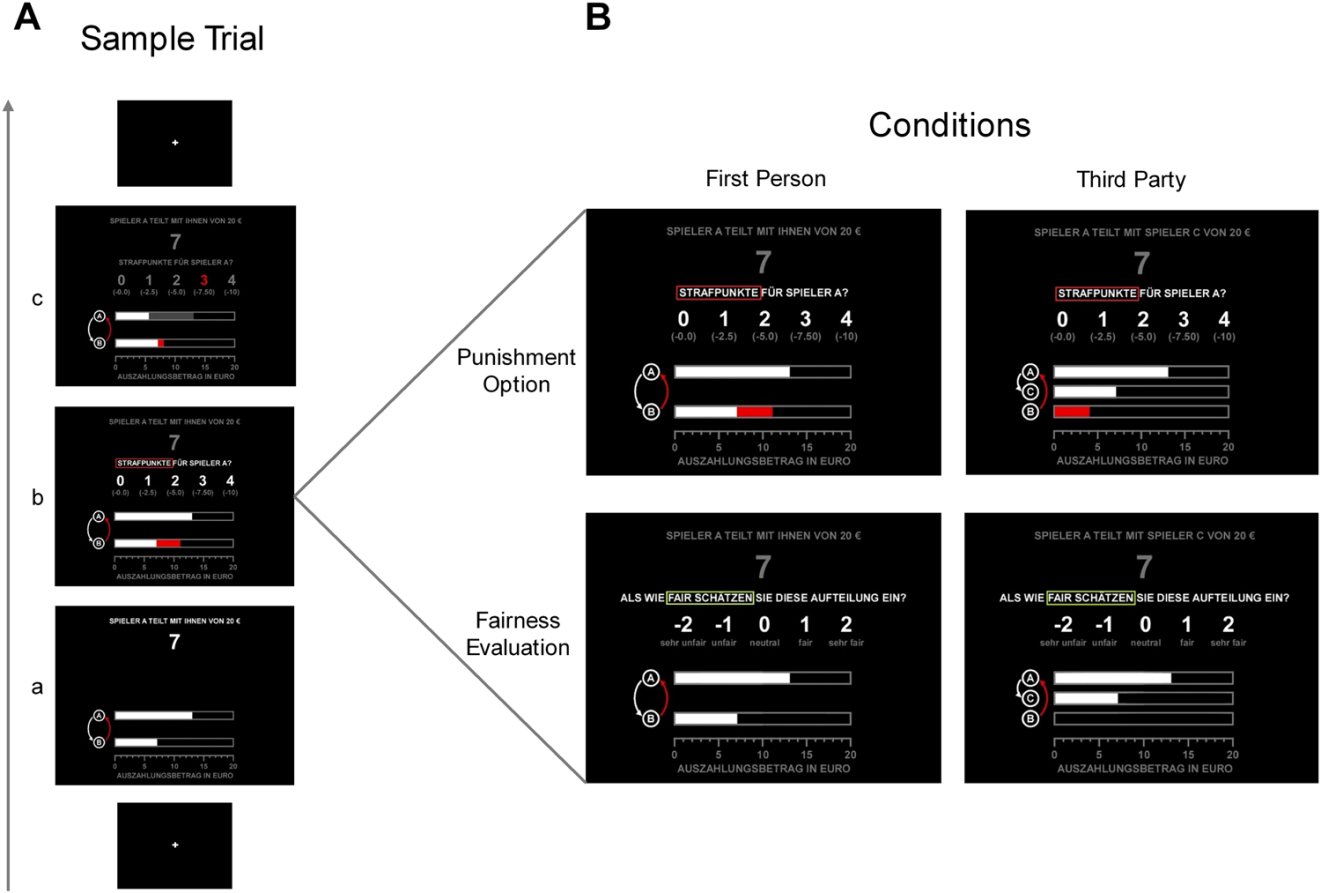
Summary of the paradigm. (**A**) Dictator Game sample trial (13:7 assignment) with punishment option in the first person condition. After viewing a fixation cross for 2,250 ms (variable duration, range 2,000–2,500 ms), (a) participants saw a specific dictator assignment for 2,000 ms (in this example 7 €). (b) Thereafter, participants had the option to punish the dictator for this assignment by allocating 0 to 4 punishment points (maximal decision time 5,000 ms). (c) After the decision, participants saw a feedback screen presenting the trial-specific outcomes for all involved parties (2,000 ms + (5,000 ms–decision time)). (**B**) Overview over the conditions used in this experiment: First person perspective/punishment option, first person perspective/fairness evaluation, third party perspective/punishment option, third party perspective/fairness evaluation.

During each of the DG runs in first person and third party scenario, participants encountered 100 assignments on computer screen. In each assignment, an anonymous dictator determined how to split 20 € between his or herself and a participant (in first person perspective scenario) or a third person (in third party perspective scenario). Each monetary assignment of the dictator constituted one trial (see Fig. 2A for a detailed trial description). Within each perspective (100 trials), in 50 trials participants had the opportunity to punish the dictator for an unfair assignment by allocating 0 to 4 punishment points (see Fig. 2B), in the remaining 50 trials, participants were able to evaluate the fairness of the current assignment (scale: −2 = very unfair to 2 = very fair). Trials regarding fairness assessment and punishment were pseudo-randomized within first person and third party perspective blocks. Table 1 displays the total distribution of dictator: recipient assignments (in €) for both the first person and third party perspectives.

**Table 1:** Total distribution of dictator–recipient assignments (in €).

Assignment	First Person	Third Party
05:15	—	1
07:13	1	—
08:12	1	—
09:11	—	1
10:10	22	32
11:09	2	3
12:08	17	10
13:07	7	3
14:06	3	6
15:05	26	13
16:04	—	4
17:03	3	4
18:02	2	3
20:00	16	20
Σ	100	100

Numbers represent the total distribution of specific dictator: recipient assignments along with the frequency in the respective perspective.

We obtained dictator assignments in advance of the actual experiment from students (*N* = 131) who were asked how they would allocate 20 € between themselves and another anonymous study participant or a third uninvolved person knowing that they could be punished by other players that would lead to a reduction of their final payoff (see also ref. 17, for more details). According to classifications from earlier studies^7, 61^, assignments less than 7 € out of 20 € were categorized as unfair, the remaining ones as fair. Each condition (i.e., fairness evaluation and punishment option within each perspective) included 25 unfair and 25 fair trials, which were randomly distributed across the conditions.

Participants were informed before the DG runs that dictators were real persons who had participated earlier and would be compensated according to their decisions. Specifically, we briefed participants that each assigned punishment point would reduce the payoff of the respective dictator by 2.50 € (e.g., resulting in a total reduction of 10 € for 4 assigned punishment points). Furthermore, we instructed participants that each withheld punishment point would benefit their compensation for participation in the study: They would receive the average dictator assignment (e.g., 5 €) plus twice the amount of average withheld punishment points (e.g., 2.7), each punishment point worth 1 € (e.g., 2 × 2.7 × 1 €).

### EEG Recording and Pre-Processing

**Figure 3:**
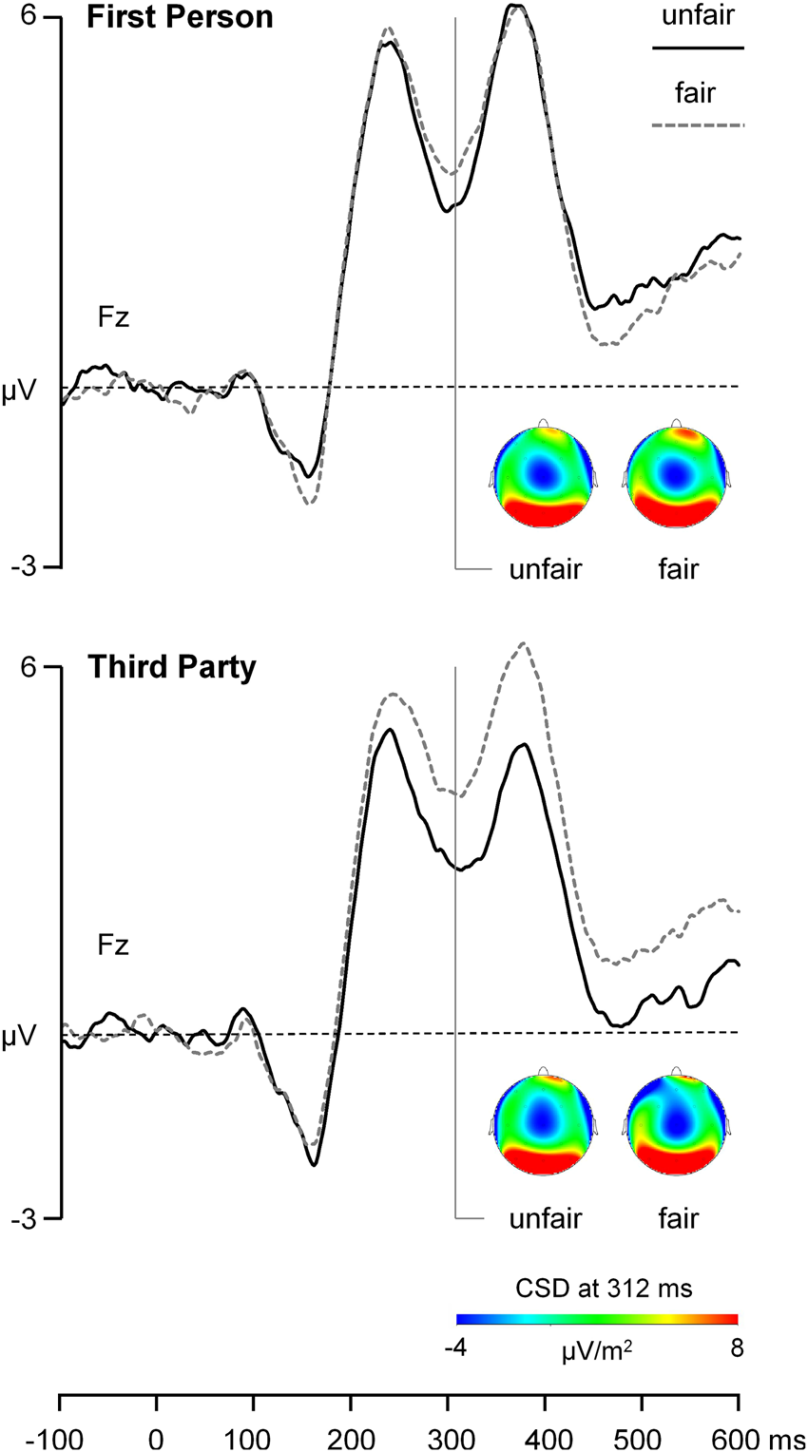
The effect of fairness on Feedback Related Negativity (FRN) and their topography after presentation of unfair and fair assignments. FRN elicited by unfair (solid black line) and fair assignments (dashed grey line) at electrode position Fz for first person (upper panel) and third party perspective (lower panel), together with topographic current density (CSD) maps at the peak FRN amplitude over conditions.

Using Brain Vision Recorder 1.3 (Brainproducts GmbH, Munich, Germany), we continuously recorded EEG, horizontal electrooculogram (HEOG), and vertical electrooculogram (VEOG) with a sampling rate of 500 Hz from 32 Ag/AgCl electrodes, which were fixed to an electrode cap (EASYCAP GmbH, Hersching, Germany) and arranged according to the enhanced 10–20 system. Left and right mastoids served as a reference and AFz as ground. We kept impedances below 5 kΩ and filtered data with a bandpass of 0.1–30 Hz. Using Brain Vision Analyzer 2.0, we segmented continuous EEG from −200 to 1800 ms after presentation of the individual assignments into the epochs “fair assignments” and “unfair assignments”. Then, we corrected ocular artifacts using the algorithm by Gratton and Coles and submitted epochs to automatic artefact detection. Channels exceeding ± 100 μV were selected as artefactual and rejected from averaging, resulting in less than 10% rejected trials on average. Epochs were baseline-corrected using the pre-stimulus interval −200 to 0 ms and then averaged separately for each participant and each experimental condition. For further FRN analyses, we used mean amplitudes from the Fz electrode in the 270–330 time window, as inspection of grand mean averages for fair and unfair trials suggested that the FRN reached its maximum about 300 ms after stimulus onset over multiple frontal electrodes located around Fz (Fig. 3).

### Statistical analyses

All analyses were performed using SPSS Statistics 21 (IBM Germany, Ehningen, Germany). To test our hypotheses on the role of dopaminergic and serotonergic gene variants in the modulation of FRN and punishment behavior, two mixed-design analyses of variance (ANOVAs) were conducted. In these models, the two variables Fairness (fair vs. unfair trials) and Perspective (first vs. third person perspective) were included as within-subject factors. *DRD4* (7+ vs. 7−) and 5-HTTLPR (l/l vs. s+) were entered as between-subject factors.

Before we conducted the mixed-design ANOVAs, correlation analyses were performed using Spearman rank correlations to examine whether the dependent and/or independent variables were related to potentially confounding factors such as sex, participants’ subjective financial situation, sleep duration as well as nicotine, caffeine and alcohol consumption. We included those variables that were identified as potentially confounding factors as covariates in the mixed design ANOVAs (see Results section).

Finally, we determined by means of Spearman rank correlations for both perspectives whether the genotypic groups differed in their individual fairness norm, that is, the hypothetical assignments that were evaluated as neither fair nor unfair by them. Higher individual fairness norms were associated with increased AP behavior (see ref. 17). Thus, we were interested in genotype-related differences with respect to individual fairness norms as they could mediate the hypothesized relationship between genotype and AP behavior. Furthermore, by means of descriptive data on individual fairness norm we further evaluated whether our categorization of assignment of less than 7 € out of 20 € as unfair and the remaining ones as fair as gained from the literature^7, 61^ was in accordance with our data.

## Results

### Potentially confounding factors

Nonparametric Spearman correlation analyses revealed a significant correlation between subjective financial situation and FRN amplitudes across fair and unfair assignments during FP perspective, *ρ* = −0.35, *p* = 0.017. Additionally, AP averaged across the two perspectives was significantly related to financial situation, *ρ* = 0.33, *p* = 0.027. This indicates that a better financial situation leads to larger FRN amplitudes and more AP. Moreover, there was a trend of carriers of the *DRD4* 7-repeat allele to be more likely male than female compared to 7-repeat non-carriers (*p* = 0.066). None of the other variables were significantly associated with the independent and/or dependent variables (all *p* > 0.10). Consequently, we controlled for participants’ sex and subjective financial situation in the mixed-design ANOVA models examining genotype-related effects on FRN and punishment behavior.

### Genotype-related effects on FRN

**Figure 4:**
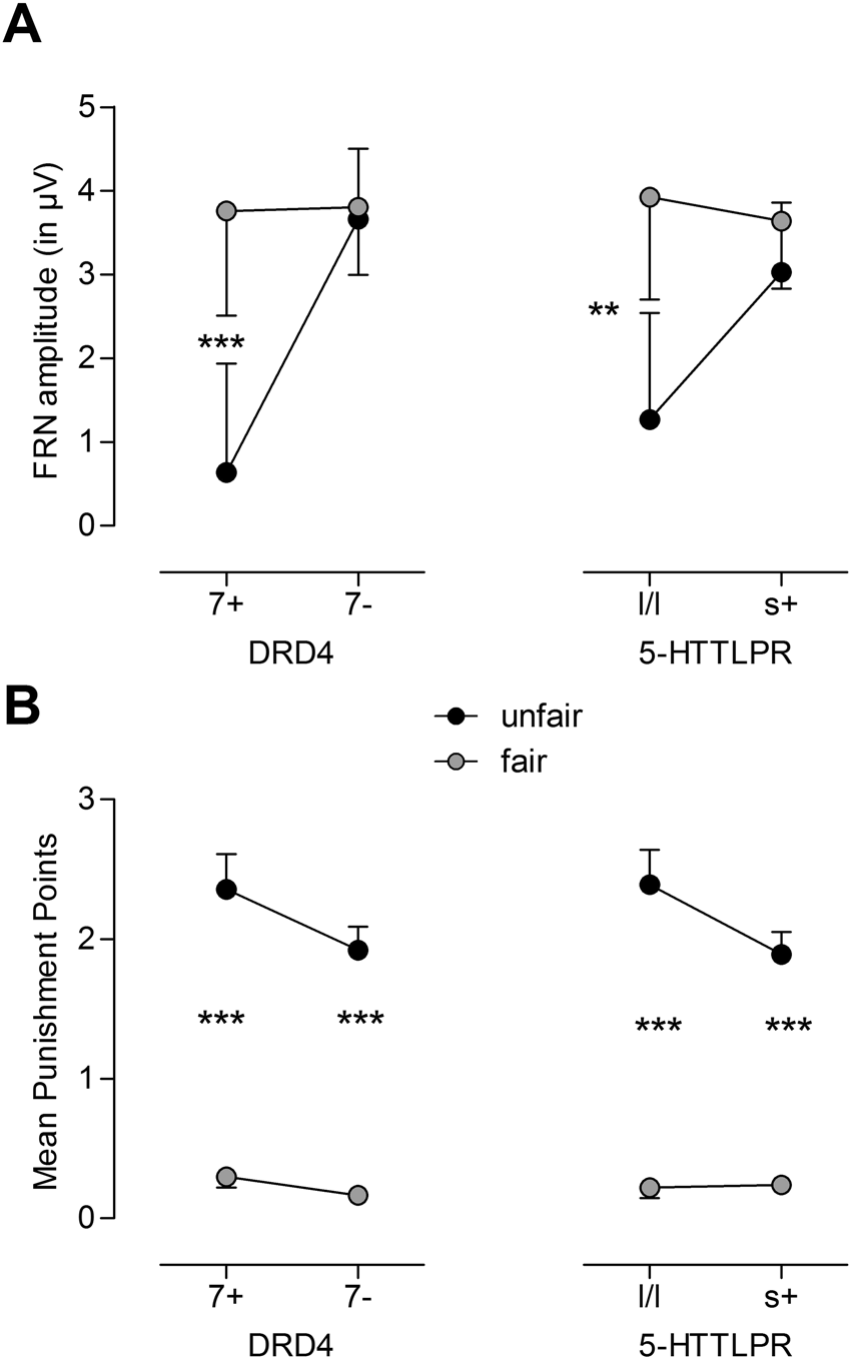
(**A**) Significant interactions of Fairness (unfair vs. fair) × *DRD4* (*F*_1,37_ = 8.83, *p* = 0.005, partial *η* ^2^ = 0.19, left panel) and Fairness × 5-HTTLPR (*F*_1,37_ = 4.47, *p* = 0.041, partial *η* ^2^ = 0.11, right panel) on FRN amplitude in the Dictator game. (**B**) Interactions of Fairness × *DRD4* (*F*_1,37_ = 1.36, *p* = 0.250, partial *η* ^2^ = 0.04, left panel) and Fairness x 5-HTTLPR (*F*_1,37_ = 4.31, *p* = 0.045, partial *η* ^2^ = 0.10, right panel) on mean punishment points in the Dictator Game; SEM of unfair (fair) trials are depicted above (below) the mean; n_DRD4_7+ = 16, n_DRD4_7− = 28, n_5-HTTLPR_l/l = 15, n_5-HTTLPR_s+ = 28; **p < 0.01, ***p < 0.001.

The mixed-design ANOVA model with FRN amplitude as dependent variable (see Fig. 3 for a graphical representation of FRN grand averages) revealed a highly significant Fairness x *DRD4* interaction with relatively large effect size, *F*(1,37) = 8.83, *p* = 0.005, partial *η* ^2^ = 0.19. As indicated by simple effect tests, larger (i.e., more negative-going) FRN amplitudes for unfair than for fair trials were observed in individuals with the 7-repeat allele (*p* < 0.001), whereas 7-repeat non-carriers did not show this differentiation (*p* = 0.785; see Fig. 4A, left panel). Furthermore, a significant Fairness x 5-HTTLPR interaction occurred, *F*(1,37) = 4.47, *p* = 0.041, partial *η* ^2^ = 0.11. In accordance with our hypothesis, l/l-genotype carriers demonstrated significantly larger FRN amplitudes for unfair than for fair dictator assignments (*p* = 0.002), while the FRN amplitude of s-allele carriers did not significantly differ between fair and unfair trials (*p* = 0.259) (see Fig. 4A, right panel).

There was no significant interaction with Perspective (all *p* > 0.10). Thus, genotype-related effects of both DRD4 Exon III and 5-HTTLPR occurred independent of whether punishment decisions were made from a first or a third party perspective. For an overview of effects, please see the Supplementary Table S1.

### Genotype-related effects on AP behavior

Next, a mixed-design ANOVA with punishment behavior as dependent variable was conducted (for all effects, see Supplementary Table S1). As expected, unfair trials were punished far more strongly than fair trials, as indicated by a highly significant Fairness main effect of large effect size, *F*(1,37) = 8.94, *p* = 0.005, partial *η* ^2^ = 0.20. Moreover, similar to the FRN results, there was a significant Fairness x 5-HTTLPR interaction, *F*(1,37) = 4.31, *p* = 0.045, partial *η* ^2^ = 0.10, suggesting that although both 5-HTTLPR groups punished unfair trials more strongly than fair trials (all *p* < 0.001), the difference in punishment to unfair compared to fair trials was more pronounced for individuals with the l/l genotype than for individuals possessing one or two copies of the s-allele (see Fig. 4B, right panel). The Fairness x *DRD4* interaction did not reach significance, *F*(1,37) = 1.36, *p* = 0.250, partial *η* ^2^ = 0.04, individuals with the 7-repeat allele only showed descriptively larger punishment differences between fair and unfair trials than non 7-repeat ones (see Fig. 4B, left panel). Moreover, a significant main effect of the subjective financial situation occurred that was further qualified by an interaction with Fairness, *F*(1,37) = 7.42, *p* = 0.010, partial *η* ^2^ = 0.17, indicating that a better financial background leads to more punishment behavior especially in unfair trials.

#### Individual fairness norm as potentially mediating factor

Finally, we examined whether individual differences in the individual fairness norm could be a mediating factor of the genotype-related influence on FRN and AP behavior. However, there were no significant associations between the genotype variables and fairness evaluation in the FP and TP perspective (all *p* > 0.10) precluding such a mediating role of individual fairness norm. Descriptive data of individual fairness norms for the different genotypic groups and perspectives can be found in Table 2. On average, individuals considered an assignment of 7 € out of 20 € as a neutral assignment (6.98 € in first person perspective, 7.08 € in third party perspective), which supports our categorization of trials with assignments of less than 7 € as unfair trials (see above).

**Table 2:** Descriptive data for individual fairness norms (in €).

Perspective	Σ	DRD4		5-HTTLPR	
		7+	7−	l/l	s+
First person	6.98 ± 1.40	6.85 ± 1.01	7.05 ± 1.61	6.87 ± 1.47	7.10 ± 1.36
Third party	7.08 ± 1.31	6.90 ± 1.19	7.22 ± 1.40	7.07 ± 1.39	7.12 ± 1.25

Data are presented as mean ± standard deviation and represent individual fairness norms for a hypothetical dictator assignment that is considered neither fair nor unfair by the average participant in the respective perspective.

## Discussion

In the present study, we addressed the role of genetic variation in key system components of DA (*DRD4* Exon III) and 5-HT (5-HTTLPR) in AP. Specifically, we focused on the influence of these genetic variations on FRN amplitude and the behavioral outcomes of recipients in response to unfair versus fair monetary assignments in a DG. As far as we know, this is the first study addressing the role of genetic variation in AP and its underlying neurophysiological correlates (i.e., FRN amplitude).

### The role of DRD4 Exon III in altruistic punishment

In support of our predictions, we found that *DRD4* exon III 7-repeat carriers showed significantly larger FRN amplitudes in response to unfair monetary assignments relative to fair ones, while non-carriers (7R-) did not. Further, 7-repeat carriers also showed descriptively larger punishment behavior in unfair trials than 7-repeat non-carriers; however, this difference missed significance. With regard to FRN, the observed association of *DRD4* with FRN amplitude is in line with immunohistochemical, northern blot, and ligand analyses, demonstrating that D4 receptors are abundantly expressed in frontal cortex regions, including the ACC^62^. Converging evidence from EEG source localization analyses and functional imaging data indicates that the FRN originates in the ACC, most likely in the dorsal ACC^63, 64^. Along with the expression pattern of D4 receptors in the ACC, previous reviews on DA function conclude that genetic variation in the D4 receptor gene may likely be associated with performance monitoring and feedback-based processing contributing to FRN modulation^57^. Thus, in line with the impact of DA signaling on FRN-related feedback processing, our results provide first evidence for an association of *DRD4* exon III and FRN amplitude. Our results also concur with previous data of Krämer, *et al*.^65^ who found another polymorphism in the *DRD4* gene (*DRD4*–521 C/T) to predict variability in the error-related negativity (ERN) during a flanker task. In this study, carriers homozygous for the T-allele, which is suggested to result in reduced D4 receptor density in frontal brain areas (similar to functional effects of the 7-repeat of *DRD4* exon III), showed increased ERN amplitudes for flanker errors and a higher number of failed inhibitions to intermixed stop-trials (i.e., larger impulsivity) relative to C-allele carriers (see also ref. 66). Thus, these data point to a relationship between D4 receptor density and electrophysiological and behavioral outcomes similar to those expected for the 7-repeat of *DRD4* exon III. Because it is assumed that ERN and FRN share overlapping neuronal and functional processes^14, 67^, this finding may provide support for the observed association of the 7-repeat allele with increased FRNs to negative feedback.

Furthermore, according to the theory of Holroyd and Coles^14^, the ACC acts as a comparator evaluating whether feedback deviates from expectations. Negative prediction errors are thought to be accompanied by phasic dips of DA signaling that are conveyed to ACC via the striatum, thereby leading to more negative-going FRN amplitudes^14, 18, 65^. Our results may be consistent to recent results suggesting an increased striatal reactivity in 7-repeat carriers, which has been assumed to augment phasic DA dips to negative outcomes/feedback^57, 65, 66, 68^. Thus, this presumably higher striatal activity could contribute to more pronounced FRN amplitudes in response to unfair monetary offers, as observed in our study. Specifically, in addition to 7-repeat-dependent reduced receptor binding and lower postsynaptic D4 receptor expression patterns in frontal brain regions^40, 69^, data from DRD4 knock-out mice suggests an increase of DA synthesis^70^. Neuroimaging studies in humans point to similar results by demonstrating increased reactivity of striatal neurons in 7-repeat carriers compared to non-carriers^68, 71^. Interestingly, this increased striatal activity in 7-repeat carriers was also correlated with higher levels in self-reported impulsivity during a card guessing game^68^, supporting other results on the relationship between striatal activation and behavioral measures of impulsivity e.g. ref. 72. Such a higher responsiveness to aversive outcomes or negative feedback, respectively, may also be linked with a relatively stronger impulse to punish unfair monetary dictator assignments at the behavioral level^19^.

Indeed, accumulating evidence shows that 7-repeat carriers are more prone to impulsive behavior, which in turn is considered an important moderator of AP^9, 25, 73^. Their less responsive D4 receptors have been argued to contribute to impulsivity-related phenotypes^69^. In fact, 7-repeat carriers have repeatedly been associated with reward and impulsivity-related behavior and personality traits such as impulsivity^44^, novelty seeking^42, 43^, behavioral disinhibition^74^, pathological gambling and (financial) risk taking^39, 45, 46^. While meta-analyses cast some doubt on the role of the *DRD4* exon III 7-repeat allele in impulsive traits^47, 48^, meta-analytic evidence consistently support the role of DRD4 exon III as a substantial risk factor for ADHD^35, 49–51^. Further, behavioral results from Ultimatum games^6, 75^ and questionnaire-based personality data of our own research^53^ suggest a relationship between the *DRD4* 7-repeat allele and lower altruism or prosocial behavior, which may also relate to their higher impulsivity. However, in the present study there were only descriptive (but not statistically significant) differences in altruistic punishment between 7-repeat carriers and non-carries. Because genetic effects on behavioral measures are typically small, the power to detect significant differences for punishment behavior may have been limited (see also limitation section).

### The role of 5-HTTLPR in altruistic punishment

Besides *DRD4* gene variation, we further addressed the potential impact of 5-HTTLPR in AP, given the prominence of serotonin in modulating neuronal and behavioral responses to aversive and punishment signals and its implication in impulsive behavior^26, 28, 31^. Accordingly, recent theorizing on feedback processing proposes serotonergic signaling to critically modulate responses to negative outcomes and thereby to code for negative prediction errors in particular^26, 59^. Indeed, in the present study, the expected relationship of 5-HTTLPR and AP was demonstrated by associations with both FRN amplitude and punishment behavior to unfair dictator assignments. In terms of FRN, as assumed, carriers homozygous for the l-allele showed larger amplitudes to unfair relative to fair monetary assignments, suggesting that they are more responsive to unfair behavior of others, while s-allele carriers did not show such a differentiation.

This presumably higher responsiveness to signals of unfairness may relate to the role of serotonin in modulating impulsive responding such as elicited by provocation, aggression or anger that have been shown to predict AP^25, 28^. Specifically, our results may be explained by evidence of studies and meta-analytic data pointing to a relatively increased proneness for impulsive tendencies in homozygous l-allele individuals^35, 38, 76, 77^. Thus, their higher sensitivity to signals of unfairness may be indexed by increased neurophysiological responses to unfair offers as well as subsequent punishment behavior. However, beyond this more general point, a more specific aspect of impulsive behavior could have also contributed to the observed effects. Several studies have consistently reported a higher financial risk taking behavior in l-allele individuals, while in contrast s-allele carries of 5-HTTLPR have been found to be more risk averse during economic decision making^38, 39, 78, 79^. Given that financial risk taking refers to the expectation to obtain monetary gains, increased FRN amplitudes (and later punishment behavior) may be due to the fact that based on unfair dictator decisions a possibly expected monetary reward cannot be obtained. According to FRN theory, such violated expectations could have elicited more pronounced FRN amplitudes in l/l-genotype carriers. Proceeding from the model of Holroyd and Coles^14^, negative feedback signals may be used to adjust the receiver’s behavior, with the aim that unfair dictator assignments may be less likely obtained in the future. Indeed, as demonstrated by our behavioral data, homozygous l-allele carriers punished unfair dictator assignments significantly more strongly than s-allele ones. In contrast, the s-allele has been associated with higher scores in neuroticism, harm avoidance, and risk aversion^30, 36, 37^. This may explain why s-allele carriers were comparatively less inclined to punish in our study than those possessing the l/l genotype: Punishing unfair behavior of others may lead to negative consequences for the punisher or would at least be perceived as a decision under ambiguity and risk.

In addition, another interpretation can be provided that is more directly focused on the role of emotions in outcome expectations: Given that the presence of the s-allele is associated with a higher likelihood to exhibit negative emotions, s-allele carriers might have a more pessimistic perspective (or less positive one) and thus, might more likely expect to receive unfair feedback than l/l-genotype carriers. In the light of FRN theory, this would reduce the likelihood of a negative prediction error to occur, because the obtained feedback does not or not strongly deviate from expectations, which results in a relatively diminished FRN deflection. This notion is supported by several recent studies that found reduced FRNs in individuals with high relative to low trait anxiety after obtaining negative outcomes in risky decision making tasks^80, 81^ and may further be supported by a similar relationship between depression and FRN amplitude^82^. In contrast, homozygous l-allele carriers may have been more optimistic in generating expectations and thus, may less likely expect to receive unfair assignments, which could be reflected in increased FRN amplitudes to unfair compared to fair trials. Indeed, individuals possessing a more optimistic bias in their expectations showed larger FRN amplitudes in response to feedback that violated their expectations^83^. Similar to our interpretation, this was argued to reflect differences in outcome expectation rather than actual outcome evaluation. Although we could not directly test this prediction, our recent data in the same sample demonstrated that positive affect was associated with a stronger punishment of unfair assignments^17^.

### Limitations and future research

Although our sample size can be considered comparatively large in the context of FRN analyses, it is rather small with regard to the genetic effects. Thus, consideration of power issues is essential. Our sample size of N = 45 enabled us to detect effects of around 5% explained variance at a significance level of 0.05 and a power of 80%. However, these calculations were done with G*Power 3.1^84^, which–like most other tools for power analysis–assumes equal cell sizes for ANOVA calculations. When repeating the analysis by doubling the smallest cell size (n = 15 for the 5-HTTLPR l/l group), the power in the resulting hypothetical sample size of N = 30 to detect an effect of 5% explained variance would drop to 0.57. Nevertheless, even 5% explained variance may be realistic especially for biological variables like the FRN that can be conceived of as being more proximate to gene action, a view inherent to the so-called endophenotype approach (e.g. ref. 85). Indeed, the variance portions in FRN explained by the genetic variables were altogether larger (19%, and 11%, respectively, for DRD4, and 5-HTTLPR, respectively) than those for punishment behaviour (4%, and 10%), and hence, our power may have been a limiting factor with regard to punishment behavior. Thus, future studies should employ larger samples that take into account (1) the frequency of the minor allele or genotype and (2) the likely smaller effect sizes to be assumed for behavioral or self-report data.

Furthermore, there is an imbalanced distribution of males and females in our study, which may have influenced the results. However, we observed no sex main or interaction effect in the present data. Moreover, future studies on *DRD4* exon III and 5-HTTLPR may focus on the possible role of specific sources of variance that could have influenced FRN amplitude and punishment behavior: While in the light of FRN theory, it is plausible that fairness violation of others (i.e., unfair monetary assignments) can trigger FRN amplitude and punishment behavior, the potential role of reward expectations that could be violated by unfair assignments should be taken into account in future studies (i.e., genotype-dependent differences in financial risk taking or loss aversion). Furthermore, recent views of reinforcement learning and feedback processing propose that the release of phasic serotonin may act as a motivational opponent to DA signaling, especially when negative feedback comes into play^26, 59, 67^. It may be conceivable that our 5-HTTLPR results on FRN amplitude reflect an effect of 5-HTTLPR-related differences in serotonin availability on phasic dopamine activity. However, there is no evidence of interactions between 5-HTTLPR and *DRD4* exon III in our data. Nonetheless, this might not preclude interactions between 5-HTTLPR genotypes and other dopamine system constituents not addressed in the present study. Thus, serotonin-dopamine interactions and the presumed role of serotonin as an opponent to DA signaling should be further addressed in future studies.

Last but not least, future studies should investigate the role of participants’ financial situation in altruistic punishment scenarios and associated neurophysiological correlates such as the FRN. In this study, larger FRN amplitudes in the face of a better financial situation of participants suggest that expectation violations due to unfair assignments may be stronger in individuals in a relatively better financial situation. Furthermore, the finding that an improved financial situation was associated with stronger altruistic punishment suggests that individuals with a better financial background might be less concerned about the cost of punishment acts (see also ref. 17).

## Electronic supplementary material

Supplementary Information
