# Supplementary material for: Genetic variation of dopamine and serotonin function modulates the feedback-related negativity during altruistic punishment
Source: Sci Rep. 2017 Jun 7;7:2996. doi: 10.1038/s41598-017-02594-3 (PMC5462809; doi:10.1038/s41598-017-02594-3)
Supplement: Supplementary file 1 — Supplementary Information [file 41598_2017_2594_MOESM1_ESM.pdf]

Supplementary material

Title: Genetic variation of dopamine and serotonin function modulates the feedback-related negativity during altruistic punishment

Authors: Enge, Mothes, Fleischhauer, Reif, & Strobel

Supplementary Table S1. Main and interaction effects of within-subject factors, between-subject factors and covariates on FRN amplitude

	df	*F*	*p*	η_p^2
Main effects of the between-subject factors and covariates				
Sex	1	1.072	.307	.028
Subjective financial situation	1	3.550	.067	.088
5-HTTLPR	1	.270	.606	.007
DRD4	1	1.105	.300	.029
5-HTTLPR × DRD4	1	.002	.961	.000
Error	37			
Main effects of the within-subject factors and interactions with the between-subject factors and covariates				
Perspective	1	.600	.444	.016
Perspective × Sex	1	.025	.875	.001
Perspective × Subjective financial situation	1	1.880	.179	.048
Perspective × 5-HTTLPR	1	.238	.628	.006
Perspective × DRD4	1	.814	.373	.022
Perspective × 5-HTTLPR × DRD4	1	.172	.680	.005
Error(Perspective)	37			
Fairness	1	1.073	.307	.028
Fairness × Sex	1	1.406	.243	.037
Fairness × Subjective financial situation	1	.772	.385	.020
Fairness × 5-HTTLPR	1	4.473	.041	.108
Fairness × DRD4	1	8.825	.005	.193
Fairness × 5-HTTLPR × DRD4	1	.605	.442	.016
Error(Fairness)	37			
Perspective × Fairness	1	.858	.360	.023
Perspective × Fairness × Sex	1	.064	.801	.002
Perspective × Fairness × Subjective financial situation	1	1.794	.189	.046
Perspective × Fairness × 5-HTTLPR	1	.059	.809	.002
Perspective × Fairness × DRD4	1	1.419	.241	.037
Perspective × Fairness × 5-HTTLPR × DRD4	1	.486	.490	.013
Error(Perspective×Fairness)	37			

Note. Gray shaded cells = statistically significant effects.

Supplementary Table S2. Main and interaction effects of within-subject factors, between-subject factors and covariates on punishment behavior

	df	*F*	*p*	η_p^2
Main effects of the between-subject factors and covariates				
Sex	1	3.381	.074	.084
Subjective financial situation	1	6.573	.015	.151
5-HTTLPR	1	1.745	.195	.045
DRD4	1	2.284	.139	.058
5-HTTLPR × DRD4	1	.078	.782	.002
Error	37			
Main effects of the within-subject factors and interactions with the between-subject factors and covariates				
Perspective	1	.008	.929	.000
Perspective × Sex	1	.975	.330	.026
Perspective × Subjective financial situation	1	.001	.918	.000
Perspective × 5-HTTLPR	1	.386	.538	.010
Perspective × DRD4	1	.499	.484	.013
Perspective × 5-HTTLPR × DRD4	1	.410	.526	.011
Error(Perspective)	37			
Fairness	1	8.939	.005	.195
Fairness × Sex	1	2.286	.139	.058
Fairness × Subjective financial situation	1	7.416	.010	.167
Fairness × 5-HTTLPR	1	4.311	.045	.104
Fairness × DRD4	1	1.364	.250	.036
Fairness × 5-HTTLPR × DRD4	1	.050	.825	.001
Error(Fairness)	37			
Perspective × Fairness	1	.419	.521	.011
Perspective × Fairness × Sex	1	2.635	.113	.066
Perspective × Fairness × Subjective financial situation	1	.206	.653	.006
Perspective × Fairness × 5-HTTLPR	1	.013	.908	.000
Perspective × Fairness × DRD4	1	.875	.356	.023
Perspective × Fairness × 5-HTTLPR × DRD4	1	.309	.581	.008
Error(Perspective×Fairness)	37			

Note. Gray shaded cells = statistically significant effects.
